# Effects of conservative approaches for treating diastasis recti abdominis in postpartum women: A systematic review and meta-analysis

**DOI:** 10.1097/MD.0000000000042723

**Published:** 2025-06-06

**Authors:** Laís Campos de Oliveira, Laura Isabel Martins de Almeida, Maria Clara Fagundes Lucio, Jorge Furtado de Campos Júnior, Raphael Gonçalves de Oliveira

**Affiliations:** a Postgraduate Program in Human Movement Sciences, Universidade Estadual do Norte do Paraná, Jacarezinho, Paraná, Brazil.

**Keywords:** exercise therapy, physical therapy modalities, postpartum period, rehabilitation

## Abstract

**Background:**

Verify the effects of conservative approaches for treating diastasis recti abdominis in postpartum women.

**Methods:**

PubMed, Embase, CENTRAL, CINAHL, SportDiscus, LILACS, and PEDro were searched, until March 15, 2024. The methodological quality of randomized clinical trials (RCTs) was assessed using the PEDro scale. Meta-analyses were conducted using the mean difference (MD) between groups for interrectus distance (IRD) in millimeters (mm).

**Results:**

After screening, 34 RCTs were included in the systematic review, of which 21 presented a low risk of bias. Very low certainty evidence demonstrated significant effects for IRD reduction in favor of abdominal exercises versus no intervention (MD = -6.82 mm) and for abdominal exercises plus multi-interventions versus abdominal exercises only (MD = -3.56 mm). Subgroup analyses demonstrated that significant IRD reduction occurs only with isotonic abdominal exercises (MD = -3.78 mm) and that the best co-intervention with abdominal exercises is electrical stimulation (MD = -4.43 mm).

**Conclusion:**

Isotonic abdominal exercises, especially when combined with electrical stimulation, represent the best conservative treatment option for improving postpartum diastasis recti abdominis. However, due to the very low certainty of the evidence, these results should be interpreted with caution, and further well-designed RCTs with high methodological quality are needed to confirm these findings.

## 1. Introduction

Diastasis rectus abdominis (DRA) is characterized by the separation of the rectus abdominis muscles along the linea alba, resulting in an increased interrectus distance (IRD). This condition is caused by weakness of the muscles in the central region of the body, particularly the anterior abdominal wall.^[1]^ Its occurrence can lead to pain, a sense of abdominal instability, and a decrease in the ability to perform daily activities.^[2]^

Several factors can induce DRA, such as obesity, but gestational factors are the most prevalent.^[3]^ These include intra-abdominal pressure from increased uterine volume and hormonal changes affecting connective tissue, leading to loosening and displacement of abdominal organs.^[3]^ Multiple pregnancies, pre-pregnancy flaccidity of the abdominal muscles, and polyhydramnios (excessive amniotic fluid during pregnancy) can also exacerbate the separation of the rectus abdominis muscles.^[4]^

The prevalence of DRA is 60% at 6 months postpartum and 32.5% at 12 months. Without intervention, DRA can persist throughout life, leading to physical and emotional harm and potentially worsening in subsequent pregnancies.^[5]^ Thus, given the critical biomechanical roles of support and stabilization provided by the abdominal wall, it is essential to develop effective strategies for the proper management of the DRA.^[6]^

To evaluate and diagnose the DRA, the IRD measurement is employed. Various assessment methods, such as palpation and calipers, are commonly used; however, two-dimensional ultrasound is the most reliable.^[7]^ During the assessment, it is essential to determine whether the separation of the rectus abdominis is partial (occurring in specific portions of the linea alba) or complete (spanning the entire length of the linea alba). Typically, the segments are characterized as supra-umbilical, umbilical, and infra-umbilical.^[8]^

For women with DRA following childbirth, exercises to strengthen the abdominal and core muscles are routinely recommended as part of the rehabilitation process. Other common conservative approaches include postural training, the use of elastic straps or bandages, and respiratory or hypopressive exercises, among others. However, there is currently no consensus in the literature regarding the most effective conservative treatment option for improving DRA after pregnancy.^[1,6,9]^

Although current systematic reviews with meta-analyses have been published on the topic,^[1,6,9]^ the most comprehensive one included only 16 randomized clinical trials (RCTs).^[6]^ Overall, these reviews identified abdominal exercises as the most promising approach. However, the limited number of RCTs included in these reviews did not allow for detailed subgroup analyses to determine, for example, whether the type of abdominal exercise (isometric or isotonic) influences the outcomes or which type of therapeutic co-intervention can be used alongside abdominal exercises to enhance results. Recently, numerous new RCTs have been published, highlighting the need for an updated systematic review.

Therefore, the objective of the present systematic review and meta-analysis of RCTs was to verify the effects of conservative approaches used to treat DRA in postpartum women.

## 2. Methods

The present study was registered with PROSPERO (CRD42024519630). For the development of methodological aspects, the recommendations of the Cochrane collaboration^[10]^ and the checklist Preferred Reporting Items for Systematic Review and Meta-Analysis Protocols (PRISMA) were followed.^[11]^

### 2.1. Inclusion criteria

Only studies that met the following criteria were included: (a) RCTs; (b) those employing conservative approaches aimed at improving DRA, such as exercise, abdominal strapping, Kinesiotaping, and electrical stimulation; (c) those evaluating the IRD; and (d) those involving post-pregnancy women.

### 2.2. Databases and search strategies

The databases searched were: PubMed, CENTRAL, SportDiscus, Embase, CINAHL, LILACS, and Physiotherapy Evidence Database (PEDro), without applying a filter that would limit the date of publications or language. The following keywords were defined for the search strategy: (“exercises” OR “kinesiotape” OR “electrical stimulation” OR “education” OR “pilates” OR “belts” OR “physiotherapy”) AND (“diastasis abdominal” OR “diastasis rectus abdominis” OR “diastasis” OR “rectus abdominis divariance” OR “diastasis rectus abdominis”) AND (“randomized controlled trial” OR “randomized controlled trial” OR “controlled clinical trial” OR “controlled trial” OR “clinical trial” OR “randomized trial” OR “randomized allocation” OR “random process” OR “placebo” OR “randomized” OR “randomized” OR “randomized” OR “study” OR “groups” OR “group” OR “allocation” OR “allocation” OR “control group” OR “control”). The searches took place on March 15, 2024.

The *PICO* method was used to structure the bibliographic search and subsequent data extraction:^[10]^ P (*population*) = postpartum women; I (*intervention*) = conservative approaches to treat DRA; C (comparison) = no intervention or other interventions designed to improve the DRA; O (*outcome*) = IRD.

### 2.3. Study selection

A reviewer conducted the initial search strategy in the databases, extracting titles and abstracts (LCO). This same reviewer then removed duplicates. Subsequently, 2 reviewers (MCFL and LIMA) independently and blindly reviewed the titles and abstracts, excluding works that did not meet the inclusion criteria. The studies that passed this phase were then read in full by the same 2 reviewers, still blind to each other’s assessments. Any unresolved disagreements between MCFL and LIMA were referred to a third party (LCO) for resolution. Additionally, a manual search was performed in the reference lists of all eligible articles and previously published systematic reviews on the topic to identify any new references.

### 2.4. Data extraction

The data extracted from each eligible study were: (a) name of the authors, year of publication and country of the study; (b) number of participants, mean and standard deviation of age in each group; (c) study duration, weekly frequency and time of each session; (d) intervention protocol; (e) other forms of intervention; (f) control group activity; (g) parity, type of birth and postpartum time; (h) form of evaluation of the IRD; (i) results. The same form for data extraction was used by 2 reviewers (MCFL and LIMA) in a blind manner, with disagreements being resolved later by consensus.

### 2.5. Assessment of the methodological quality of studies

The methodological quality of each RCT was assessed using the PEDro scale,^[12]^ using the score available in the database itself (https://pedro.org.au/).^[13]^ If a study was not classified in the PEDro database, 2 independent reviewers (MCFL and LIMA) performed the classification blindly. In cases of disagreement, a third reviewer (LCO) was consulted. The PEDro scale consists of 11 items, but the score of the first item is not included in the final score. As a result, each study receives a final score ranging from 0 to 10 points. Studies with a final score below 6 are considered to be of low methodological quality, indicating a high risk of bias.

### 2.6. Statistical analysis

For the meta-analysis, the effect measure was the mean difference between the groups at the post-intervention time for IRD measured in millimeters (mm). The Cochrane *Q* test for heterogeneity was performed and considered statistically significant if *P* ≤ .10. Heterogeneity was also quantified using the I² statistic, where 0% to 40% may not be important, 30% to 60% may represent moderate heterogeneity, 50% to 90% may represent substantial heterogeneity, and 75% to 100% is defined as considerable heterogeneity.^[10]^ Fixed effects models were used when there was no statistically significant heterogeneity; otherwise, random effects models were employed. The effects of the interventions were considered statistically significant when *P* < .05. All analyses were processed using the Review Manager (RevMan) program.

### 2.7. Assessment of the certainty of evidence

The certainty of the evidence was classified according to the Grading of Recommendations, Assessment, Development, and Evaluation (GRADE) system,^[14,15]^ by 2 independent, blinded reviewers (MCFL and LIMA), with disagreements resolved by consensus. GRADE includes domains that can lower the certainty of evidence: (a) risk of bias; (b) inconsistency; (c) indirect evidence; (d) imprecision; (e) other factors (such as publication bias). After evaluation, the final classification of the certainty of the evidence for each outcome can be: (a) high (it is unlikely that new research will change the estimate of the effect); (b) moderate (future research is likely to impact the effect estimate and may modify it); (c) low (future research is likely to have a significant impact on the effect estimate and change it); (d) very low (results are highly uncertain).

### 2.8. Subgroup analyses

In order to verify whether studies with a high risk of bias were influencing the primary analyses, subgroup analyses were separately carried out with studies of high and low methodological quality in the meta-analysis charts. Other subgroup analyses that were feasible included: (a) abdominal exercises versus control by observing isotonic and isometric exercises separately; (b) abdominal exercises versus abdominal exercises plus electrical stimulation.

## 3. Results

### 3.1. Included studies and participants

**Figure 1.**
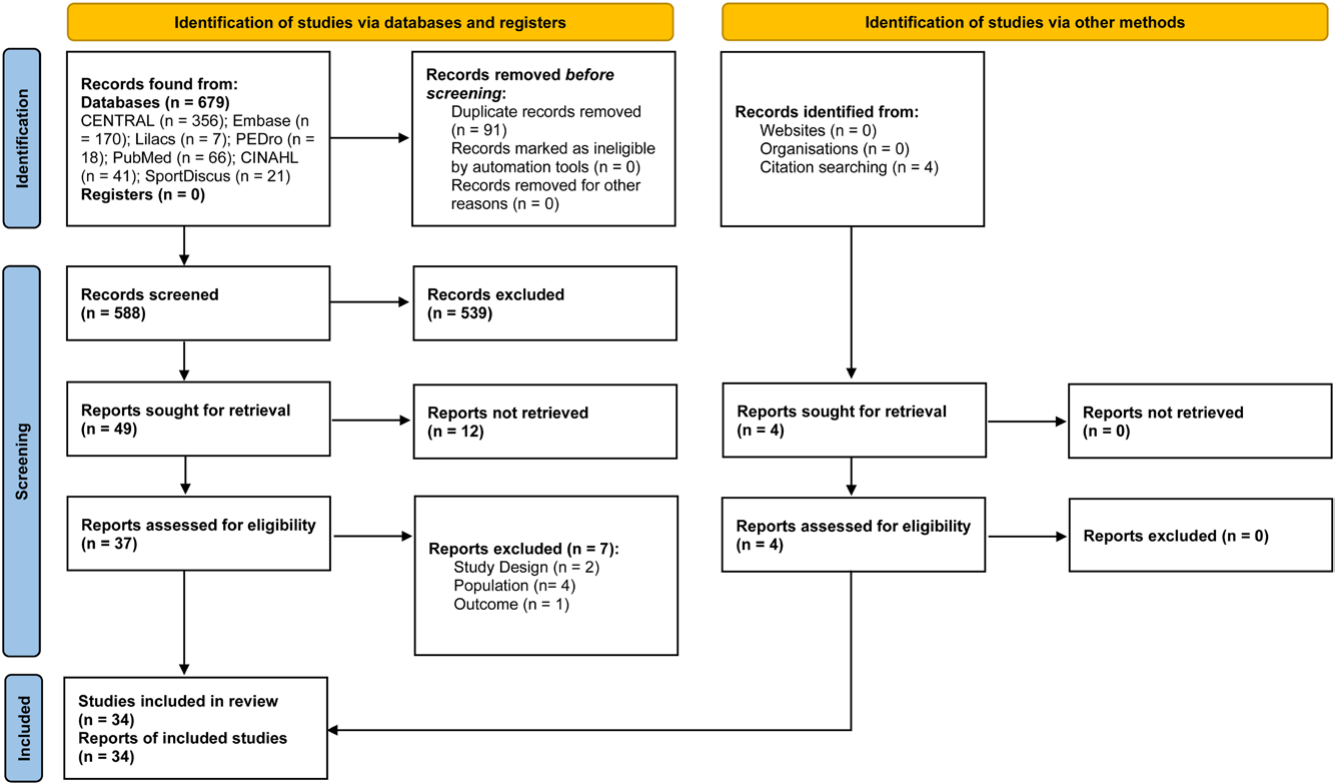
PRISMA flow diagram. PRISMA = Preferred Reporting Items for Systematic Review and Meta-Analysis Protocols.

A total of 679 records were identified in the initial search within the databases. Of these, 91 were excluded because they were duplicates. After reading the titles and abstracts, 539 were excluded because they did not meet the inclusion criteria. Thus, 49 reports remained to be searched for reading in full. Of these, 12 were not retrieved, as they were only clinical trial records or were not accessed after all forms of search and contact with the authors (Table S1, Supplemental Digital Content, https://links.lww.com/MD/P128). Finally, of the 37 studies assessed for eligibility, 2 were not RCTs, 4 did not include postpartum women, and 1 did not assess DRA (Table S2, Supplemental Digital Content, https://links.lww.com/MD/P128). Thus, 30 articles remained that initially met the inclusion criteria for this review. Subsequently, the references of all included studies and preexisting systematic reviews were accessed in order to find other potentially eligible reports. Hereby, 4 new reports met the inclusion criteria. Therefore, the present systematic review was conducted with 34 studies, represented by 1 report each. The PRISMA flow diagram illustrates the identification, screening, and inclusion events of articles (Fig. 1).

Table S3, Supplemental Digital Content, https://links.lww.com/MD/P128 presents a summary of the studies included in the systematic review. Among the 34 studies, 3 were conducted in India,^[16–18]^ 4 in Egypt,^[19–22]^ 3 in Brazil,^[23–25]^ 1 in Turkey,^[26]^ 1 in Malaysia,^[27]^ 4 in Iran,^[28–31]^ 2 in Norway,^[32,33]^ 1 in South Korea,^[34]^ 1 in New Zealand,^[35]^ 1 in Australia,^[36]^ 1 in Switzerland,^[37]^ 3 in China,^[38–40]^ 3 in the United States,^[41–43]^ 2 in Poland,^[44,45]^ 1 in Pakistan,^[46]^ 1 in Saudi Arabia,^[47]^ 1 in Canada,^[48]^ and 1 in Bahrain.^[49]^ The oldest study included was published in 1999,^[25]^ and the most recent in 2024.^[16]^

The total number of women analyzed was 1548, aged between 20 years^[17]^ and 40 years.^[25]^ The forms of assessment used in studies to measure IRD were: caliper,^[16–18,22,24,25,27–30,36,41,44,47]^ ultrasound,^[19–21,23,26,31,32,34,35,37–40,42,48,49]^ palpation method,^[33,45]^ and the joint use of 2 methods (palpation and caliper^[18,46]^ or ultrasound and caliper).^[43]^

Regarding the number of births, 14 studies included primiparous and multiparous women,^[16,18,25,26,31,32,35,38–43,45]^ 8 included only primiparous women,^[17,22,27,33,34,44,48,49]^ 5 studies covered only multiparous women,^[21,24,28–30]^ and 7 did not report parity.^[19,20,23,36,37,46,47]^ With regard to types of birth, 13 studies covered vaginal birth and cesarean section,^[16,18,23,26,32,34,35,37,40,41,43,45,46]^ 19 studies included only vaginal birth,17,^[19–22,24,25,27,28,30,31,33,36,38,39,44,47–49]^ 1 study covered only cesarean section,^[29]^ and 1 study did not specify the type of delivery.^[42]^

### 3.2. Intervention

The weekly frequency of interventions varied between 1,^[33]^ 2,^[23,37]^ 3,^[16,17,19–22,26–30,38,41,43,46,49]^ 5,^[18,32,34,42,47]^ 7,^[31,39,45,48]^ and ten times a week.^[36,40]^ Session time was 10 minutes per session in 1 study,^[32]^ 20 to 30 minutes in 9 studies,^[16,17,19,21–23,38,40,49]^ and 40 to 60 minutes in 6 studies.^[28–30,33,34,37]^ In 15 studies, session time was not reported.^[18,20,26,27,31,35,36,39,41–43,45–48]^

The time from which interventions began after birth varied from 6 hours^[24,25]^ to 3 years,^[18,43]^ with only 5 studies intervening in the immediate postpartum period (0–1 week).^[22,24,25,35,45]^ The number of intervention groups in each study varied from 2,^[16–25,27,30–41,43–47,49]^ 3,^[26,28,29]^ up to 4 groups,^[42,48]^ with the forms of interventions being quite heterogeneous, including:

1. abdominal exercises;^[16,18,21,22,26–29,31,32,34,36,37,42,45,47–49]^
2. isometric abdominal exercise plus other interventions: hypocaloric diet and resistive capacitive electrical transfer;^[19]^ use of a compressive abdominal belt;^[20,26]^ isotonic abdominal exercises plus use of the belt,^[48]^ use of the belt plus pelvic floor muscle training (PFMT);^[41]^ isotonic abdominal exercises and PFMT;^[24,25]^ PFMT;^[46]^ electrical stimulation;^[17]^ Kinesiotaping;^[17,42]^
3. isotonic abdominal exercise plus other interventions: electrical stimulation;^[38,39,49]^ PFMT plus electrical stimulation;^[38]^ PFMT, isometric abdominal and electroacupuncture;^[40]^ PFMT and isometric abdominal;^[30,40]^ PFMT;^[33,46]^ isometric abdominals plus electrical stimulation;^[21]^ isometric abdominal exercises plus strap;^[18]^ isometric abdominal exercises, use of the belt and PFMT;^[43,47]^ PFMT using the strap;^[43]^
4. other interventions, such as: Hypopressive exercises;^[16,23]^ use of a compressive abdominal belt;^[20,22,26,35,48]^ walking exercises;^[27]^ functional exercises;^[28]^ Kinesiotaping;^[35,42,44]^ PFMT;^[29]^
5. control groups.^[23–25,28–34,39,41,42,44,45,48]^

Regarding the duration of the study, there was a variation from 2^[40]^ to sixteen weeks,^[33]^ with 6^[21,22,31,36–39,43,45,46]^ and 8 weeks^[16,20,26–30,35,47,49]^ being the total intervention time most used.

It was possible to group 12 different intergroup comparisons that demonstrated significant effects in reducing post-pregnancy DRA:

1. in favor of abdominal exercises vs control groups,^[28,31,32,34,42]^ hypopressive exercises,^[16]^ or walking exercises;^[27]^
2. in favor of abdominal exercises plus compressive belt vs using only the belt,^[20,22,36]^ or performing only abdominal exercises;^[18,26,47]^
3. in favor of abdominal exercises plus electrical stimulation,^[21]^ or resistive capacitive electrical transfer vs performing only abdominal exercises;^[19]^
4. in favor of abdominal exercises plus other interventions (PFMT, Kinesiotaping, compressive abdominal strapping, electrical stimulation, functional exercises) vs control group,^[24,25,29,30,33,39,41,42,45]^ or carrying out of just abdominal exercises;^[49]^
5. in favor of isotonic vs isometric abdominal exercises;^[36]^
6. in favor of isotonic abdominal exercises plus PFMT vs isometric abdominal exercises plus PFMT;^[46]^
7. in favor of functional exercises vs control;^[28]^
8. in favor of Kinesiotaping vs control group;^[44]^
9. in favor of abdominal exercises plus PFMT and electrical stimulation vs abdominal exercises plus electrical stimulation;^[38]^
10. in favor of abdominal exercises plus electrical stimulation vs abdominal exercises plus application of Kinesiotaping;^[17]^
11. in favor of Kinesiotaping associated with abdominal exercises vs Kinesiotaping;^[42]^
12. in favor of abdominal exercises vs Kinesiotaping.^[42]^

### 3.3. Methodological quality of studies

The assessment of methodological quality, presented in Table 1, demonstrated that of the 34 studies included in the systematic review, 21 presented a low risk of bias (PEDro score ≥ 6 points). However, 13 studies were at high risk of bias (PEDro score ≤ 5). The average score across RCTs was 5.82 ± 1.38.

**Table 1:** Methodological quality of studies included in the systematic review.

Study	C1*	C2	C3	C4	C5	C6	C7	C8	C9	C10	C11	Score† (0–10)
Sekar et al (2024)^[16]^	+	+	+	+	-	-	-	+	+	+	+	7
Elhosary et al (2023)^[19]^	+	+	+	+	-	-	-	+	+	+	+	7
Moreira (2023)^[23]^	+	+	+	+	-	-	+	+	+	+	+	8
Kaya and Menek (2023)^[26]^	+	+	-	+	-	-	-	+	-	+	+	5
Shohaimi et al (2023)^[27]^	-	+	+	+	-	-	-	-	-	+	+	5
Yalfani et al (2023)^[28]^	+	+	+	+	-	-	+	-	-	+	+	6
Gluppe et al (2023)^[32]^	+	+	+	+	-	-	+	+	+	+	+	8
Lee et al (2023)^[34]^	+	+	-	+	-	-	-	+	+	+	+	6
Depledge et al (2023)^[35]^	+	+	-	+	-	-	+	+	+	+	+	7
Simpson and Hahne (2023)^[36]^	+	+	+	+	-	-	+	-	+	-	+	6
Kim et al (2022)^[37]^	+	+	-	+	-	-	-	+	+	+	+	6
Liang et al (2022)^[38]^	-	+	+	+	-	-	+	+	+	+	+	8
Wei et al (2022)^[39]^	+	+	-	+	-	-	-	-	-	+	+	4
Liu et al (2022)^[40]^	+	+	+	+	-	-	+	+	+	+	+	8
Safaee et al (2022)^[29]^	-	+	-	+	-	-	-	-	-	+	+	4
Keshwani et al (2021)^[48]^	+	+	-	+	-	-	+	+	-	+	+	6
Laframboise et al (2021)^[41]^	+	+	+	+	-	-	-	+	-	+	+	6
Pampolim et al (2021)^[24]^	+	+	-	+	-	-	-	+	+	+	+	6
Ptaszkowska et al (2021)^[44]^	-	+	-	+	-	-	-	-	-	-	+	3
Awad et al (2021)^[20]^	+	+	+	+	-	-	-	+	-	+	+	6
Saleem et al (2021)^[46]^	+	+	-	-	-	-	+	+	-	+	+	5
Situt and Kanase (2021)^[17]^	-	+	-	+	-	-	-	-	-	+	+	4
Botla and Saleh (2020)	+	+	+	+	-	-	+	+	-	+	+	7
Yalfani et al (2020)^[30]^	+	+	-	+	-	-	-	-	-	+	+	4
Dave and Mahishale (2019)^[18]^	+	+	-	+	-	-	-	+	-	+	+	5
Thabet and Alshehri (2019)^[47]^	+	+	-	+	-	-	+	+	-	+	+	6
Izardi et al (2018)^[31]^	+	+	-	+	-	-	-	+	-	+	+	5
Bobowik and Dabek (2018)^[45]^	-	+	-	+	-	-	-	-	-	+	+	4
Gluppe et al (2018)^[33]^	+	+	+	+	-	-	+	+	+	+	+	8
Tuttle et al (2018)^[42]^	+	+	+	+	-	-	+	+	-	+	+	7
Kamel and Yousif (2017)^[49]^	+	+	-	+	-	-	+	+	-	+	+	6
Walton et al (2016)^[43]^	+	+	-	+	-	-	+	+	-	+	+	6
El-Mekawy et al (2013)^[22]^	+	+	-	-	-	-	-	+	-	+	+	4
Mesquita et al (1999)^[25]^	+	+	-	-	-	-	-	+	+	+	+	5

+: criteria met; –: criteria not met; C1: eligibility criteria were specified; C2: subjects were randomly allocated to groups; C3: allocation was concealed; C4: the groups were similar at baseline regarding the most important prognostic indicators; C5: there was blinding of all subjects; C6: there was blinding of all therapists who administered the therapy; C7: there was blinding of all assessors who measured at least 1 key outcome; C8: measures of at least 1 key outcome were obtained from more than 85% of the subjects initially allocated to groups; C9: all subjects for whom outcome measures were available received the treatment or control condition as allocated or, where this was not the case, data for at least 1 key outcome was analyzed by “intention to treat”; C10: the results of between‐group statistical comparisons are reported for at least 1 key outcome; C11: the study provides both point measures and measures of variability for at least 1 key outcome.

* Item not considered in the score.

† Overall average (SD): 5.82 (1.38).

### 3.4. Quantitative synthesis of studies (meta-analysis)

For each meta-analysis, we analyzed the certainty of the evidence using the GRADE system. For the different analyses, the certainty of the evidence varied from very low to moderate, with the main problems being linked to risk of bias, inconsistency, and imprecision (Table 2).

**Table 2:** Analysis of the certainty of the evidence.

Certainty assessment					No. of patients		Mean difference (95% CI)	GRADE certainty
No. of RCTs	Risk of bias	Inconsistency	Indirectness	Imprecision	Intervention	Comparison		
Abdominal exercises versus control in postpartum diastasis recti								
7	Serious*	Serious†	Not serious	Serious‡	121	115	-6.82 mm (-12.82, -0.82)	⨁**◯◯◯** Very low
Abdominal exercises versus control in postpartum diastasis recti (at 6 hours postpartum)								
2	Serious*	Serious†	Not serious	Serious‡	50	50	-2.00 mm (-4.74, 0.73)	⨁**◯◯◯** Very low
Isometric abdominal exercise versus compressing method in postpartum diastasis recti								
3	Not serious	Serious†	Serious§	Serious‡	32	31	-1.04 mm (-2.81, 0.73)	⨁**◯◯◯** Very low
Isometric abdominal exercise plus other interventions versus control in postpartum diastasis recti								
2	Not serious	Not serious	Serious§	Serious‡	9	11	2.04 mm (-8.33, 12.42)	⨁⨁**◯◯** Low
Isometric abdominal exercise plus abdominal belt versus abdominal belt in postpartum diastasis recti								
2	Not serious	Not serious	Not serious	Serious‡	22	23	-0.00 mm (-2.57, 2.56)	⨁⨁⨁**◯** Moderate
Abdominal exercises versus abdominal exercises plus multi-interventions in postpartum diastasis recti								
8	Not serious	Serious†	Serious§	Serious‡	146	142	-3.56 mm (-4.96, -2.16)	⨁**◯◯◯** Very low

CI = confidence interval, RCT = randomized clinical trial.

* Subgroup analysis demonstrated that significant results are observed only in the grouping of studies with a high risk of bias.

† Analysis showed high heterogeneity that was not explained by subgroup analyses.

‡ Suboptimal sample size.

§ The studies included in the analysis involved different forms of intervention.

**Figure 2.**
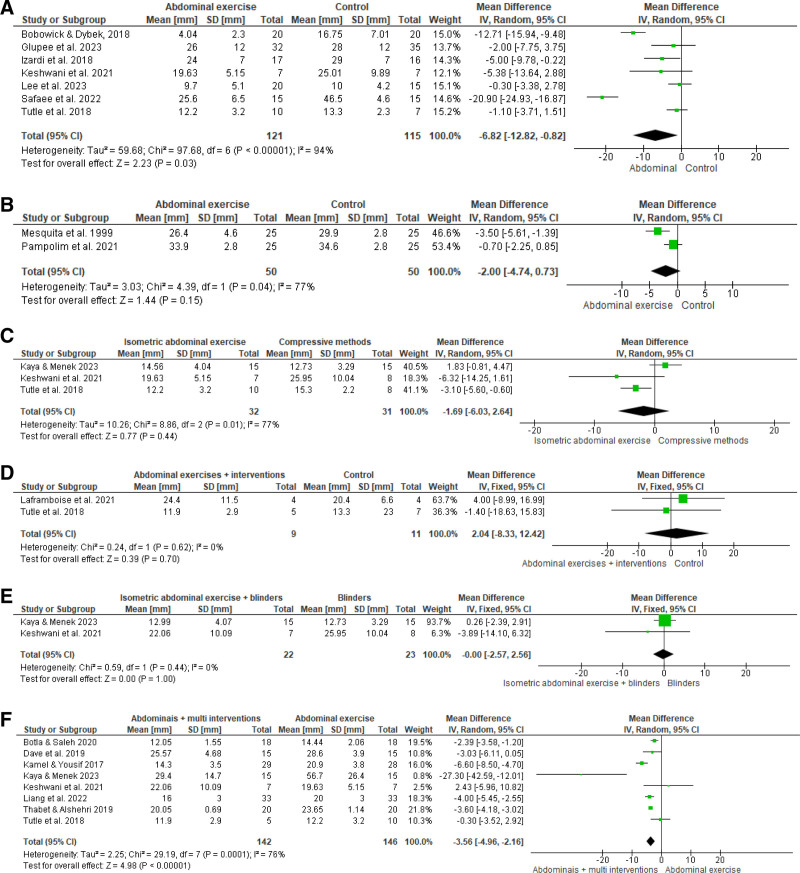
Primary analysis of the effects of comparisons on postpartum abdominal diastasis: (A) abdominal exercises versus control group; (B) abdominal exercises versus control group (6 h postnatal); (C) isometric abdominal exercises versus compressive methods (abdominal strapping and Kinesiotaping); (D) isometric abdominal exercises plus other interventions (abdominal strapping, electrical stimulation, or breathing exercises) versus control group; (E) isometric abdominal exercises plus abdominal strapping versus abdominal strapping; (F) abdominal exercises versus abdominal exercises plus multi-interventions (electrical stimulation, use of an abdominal belt, breathing exercises, PFMT, Kinesiotaping). PFMT = Pelvic Floor Muscle Training.

Figure 2 presents the primary analyses. There was very low certainty evidence (downgraded by risk of bias, inconsistency, and imprecision) that abdominal exercises are significantly superior to control groups in reducing DRA (-6.82 mm [95% CI -12.82, -0,82], *P* = .03, I^2^ = 94%, n = 236, studies = 7; Fig. 2A). When abdominal exercises were compared with control groups in the immediate postpartum period, there was very low certainty of evidence (downgraded by risk of bias, inconsistency, and imprecision) that there were no significant differences between the groups (-6.82 mm [95% CI -12.82, -0,82], *P* = .03, I^2^ = 94%, n = 236, studies = 7; Fig. 2B).

When isometric abdominal exercises were compared with compressive methods (abdominal strapping and kinesiotaping), there was very low certainty of evidence (downgraded by inconsistency, indirectness, and imprecision) that there is no significant difference (-1.69 mm [95% CI -6.03, 2.64], *P* = .44, I^2^ = 77%, n = 63, studies = 3; Fig. 2C). When isometric abdominal exercises plus other interventions (abdominal strapping, electrical stimulation, breathing exercises) were compared with control groups, there was low certainty of evidence (downgraded by indirectness and imprecision) that there is no significant difference (2.04 mm [95% CI -8.33, 12.42], *P* = .70, I^2^ = 0%, n = 20, studies = 2; Fig. 2D). Similarly, when isometric abdominal exercises plus abdominal strapping was compared with abdominal strapping, there was moderate certainty of evidence (downgraded for imprecision) that there is no significant difference (-0.00 mm [95% CI -2.57, 2.56], *P* = 1.00, I^2^ = 0%, n = 45, studies = 2; Fig. 2E).

Finally, there was very low certainty evidence (downgraded by inconsistency, indirect evidence, and imprecision) that abdominal exercises plus multi-interventions (electrical stimulation, abdominal strapping, breathing exercises, PFMT, kinesiotaping) are significantly superior to abdominal exercise in reducing DRA (-3.56 mm [95% CI -4.96, -2.16], *P* = .00001, I^2^ = 76%, n = 288, studies = 8; Fig. 2F).

### 3.5. Subgroup analysis

Figure S1, Supplemental Digital Content, https://links.lww.com/MD/P129 presents the subgroup analyses. When subgroup analyses were carried out considering studies with high and low risk of bias separately, there were no changes to what had been observed in the primary analysis, with the exception of the analysis involving studies with high risk of bias that compared isometric abdominal exercises versus compressive methods, in which significant effects began to be observed in favor of abdominal exercises. When isotonic (Figure S2, Supplemental Digital Content, https://links.lww.com/MD/P129) and isometric (Figure S3, Supplemental Digital Content, https://links.lww.com/MD/P129) abdominal exercises were analyzed separately versus control groups, significant effects were observed only in favor of isotonic exercises. Finally, when separately analyzing studies that performed abdominal exercises versus abdominal exercises plus electrical stimulation (Figure S4, Supplemental Digital Content, https://links.lww.com/MD/P129), significant results were observed in favor of the association.

## 4. Discussion

This systematic review and meta-analysis included 34 RCTs with a total of 1548 participants. The objective was to assess the effectiveness of conservative approaches in improving DRA in post-pregnancy women. Our results indicated that abdominal exercises significantly reduced IRD by -6.82 mm compared to no intervention. However, subgroup analysis showed a significant reduction in IRD (-3.78 mm) only with isotonic abdominal exercises. When comparing abdominal exercises alone to abdominal exercises combined with other interventions, the combination showed a significant effect (-3.56 mm), with the greatest reduction observed when combined with electrical stimulation (-4.43 mm). It is important to note that these analyses have very low certainty of evidence, making the results highly uncertain and subject to change with future RCTs.

Still, this study presents the best evidence available at this time regarding conservative interventions to improve DRA in post-pregnancy women. The literature has highlighted that exercising in the postnatal period provides essential physiological and psychological benefits in a woman’s life.^[6]^ Studies have shown that increased DRA has a negative impact related to anguish, fear/avoidance of movement, increased body mass index, and lumbopelvic pain, contributing to disabilities and difficulties in activities of daily living.^[50,51]^ Therefore, it is possible that isotonic abdominal strengthening exercises, preferably associated with electrical stimulation, can influence the psychosocial state, improving mood and well-being, reducing kinesiophobia, and enhancing functionality.^[2,50–53]^

In addition to conservative treatment, which was addressed in the present study, there is also the possibility of surgical interventions on the connective tissue, which can reduce IRD.^[44]^ However, surgery only unites the rectus abdominis without altering the properties of the connective tissue and muscle functionality. Furthermore, the surgical method is not financially viable for the entire population and may involve restrictions and post-surgical complications.^[6]^ In view of this, conservative treatment should be considered as the first option to reduce IRD.^[54]^

With regard to the intervention time, considering that DRA is characterized by the separation of the rectus abdominis along the linea alba, composed of connective tissue, there is a need to respect a minimum time so that significant results can be observed. A previous study demonstrated that to change the mechanical properties of connective tissue, the intervention period needs to be ≥ 12 weeks.^[6]^ However, in the present systematic review, only 1 RCT respected this period of time, intervening for 16 weeks.^[33]^ This may have negatively impacted the observation of significant IRD improvements in some of the RCTs. Furthermore, because the interventions were carried out for a short period of time, it was not possible to perform subgroup analyses considering the intervention time as a possible moderating variable.

Regarding evaluation methods, more than half of the RCTs included used validated instruments to measure post-pregnancy DRA (ultrasound and caliper), with two-dimensional ultrasound being considered the gold standard for this outcome, although its clinical use is hampered by its high cost. On the other hand, the caliper is more viable for clinical practice and is scientifically validated.^[7]^ Another method used among the RCTs was palpation, which, although efficient in screening for DRA, is not scientifically supported for measuring it, since finger measurements vary between evaluators, potentially resulting in measurement errors.^[55]^

The findings of this review corroborate previous systematic reviews with meta-analysis,^[1,6,9]^ which suggest that abdominal exercises seem to be the most promising for rehabilitating post-pregnancy DRA. However, in contrast to previous studies, this review, which updates the literature on the subject, included 18 new RCTs. Additionally, the present study distinguishes itself by carrying out subgroup analyses that identified moderating variables impacting the observed effects, such as the way abdominal exercises are performed (isotonic) and the type of therapeutic co-intervention that can be associated with these exercises (electrical stimulation) to enhance the intervention’s effects.

### 4.1. Certainty of evidence

Our analyses demonstrated a certainty of evidence ranging from very low to moderate. The main issues were associated with the risk of bias, inconsistency, and imprecision. Regarding the risk of bias, it was observed that no study blinded participants and therapists. However, as the interventions involved exercises, which are perceptible stimuli, it is practically impossible to meet these two criteria. Furthermore, of the 34 RCTs included in the review, less than half implemented blind allocation,^[16,19–21,23,27,28,32,33,36,38,40–42]^ only 15 blinded the evaluators,^[21,23,28,32,33,35,36,38,40,42,43,46–49]^ and a minority performed an intention-to-treat analysis,^[16,19,23–25,32–38,40]^ which should be considered in future studies.

Regarding inconsistency, it is worth noting that 4 of the 6 primary analyses showed high heterogeneity. This implies that the meta-analysis calculations were mostly driven by random effects, leading to inconsistency in results due to discrepancies in effect sizes observed between studies. Concerning imprecision, it is important to highlight that no meta-analysis calculations were performed with an ideal information size due to small samples in the RCTs. Only 3 of the 16 RCTs included in the meta-analyses carried out sample size calculations,^[29,34,38]^ which may have compromised the sample size of individual studies, representing a critical issue that future RCTs should consider. Finally, due to the small number of studies included in each meta-analysis (<10 RCTs), it was not feasible to analyze possible publication bias using a funnel plot.

### 4.2. Strengths and limitations

This review only included RCTs, thereby reducing the risk of bias. It is worth mentioning that there were no restrictions regarding language or year of publication during the searches. Another strong point is that this systematic review covered all conservative treatment options tested to improve DRA in post-pregnancy. However, a limitation is that the search was not conducted in all existing databases, only the main ones relevant to our outcome of interest (PubMed, EMBASE, CENTRAL, CINAHL, SPORTDiscus, LILACS, and PEDro). This limitation was mitigated by performing a detailed search of all bibliographic references of the included studies, through which we found 4 more RCTs that met the inclusion criteria.

## 5. Conclusion

### 5.1. Implications for research

The findings of this review suggest the effectiveness of abdominal exercises, especially isotonic exercises associated with electrical stimulation, in reducing DRA. However, the available evidence indicated very low to moderate certainty, requiring caution in interpreting these results. It is recommended that future RCTs address the critical points identified in this review: (a) intervention duration > 12 weeks; (b) evaluation of IRD with validated instruments; (c) larger number of participants; (d) blind allocation of participants; (e) blinded evaluators; (f) intention-to-treat analysis. Finally, it is suggested that the intervention identified as the most promising conservative treatment for improving DRA in post-pregnancy (isotonic abdominal exercises plus electrical stimulation) be tested in robust RCTs to confirm these findings.

### 5.2. Implications for clinical practice

Although the certainty of the evidence is low, our findings suggest that the most effective intervention to improve postpartum DRA in women is abdominal exercises, particularly isotonic exercises combined with electrical stimulation. Therefore, until more studies are conducted, professionals can consider using this form of intervention.

## Author contributions

**Conceptualization:** Laís Campos de Oliveira, Raphael Gonçalves de Oliveira.

**Data curation:** Laura Isabel Martins de Almeida, Maria Clara Fagundes Lucio.

**Formal analysis:** Laura Isabel Martins de Almeida, Maria Clara Fagundes Lucio.

**Investigation:** Laura Isabel Martins de Almeida, Maria Clara Fagundes Lucio.

**Methodology:** Laís Campos de Oliveira, Jorge Furtado de Campos Júnior, Raphael Gonçalves de Oliveira.

**Project administration:** Laís Campos de Oliveira.

**Resources:** Jorge Furtado de Campos Júnior.

**Supervision:** Laís Campos de Oliveira, Raphael Gonçalves de Oliveira.

**Validation:** Laís Campos de Oliveira, Jorge Furtado de Campos Júnior, Raphael Gonçalves de Oliveira.

**Writing – original draft:** Laura Isabel Martins de Almeida, Maria Clara Fagundes Lucio, Jorge Furtado de Campos Júnior.

**Writing – review & editing:** Laís Campos de Oliveira, Raphael Gonçalves de Oliveira.

## Supplementary Material


