# Supplementary material for: Effects of conservative approaches for treating diastasis recti abdominis in postpartum women: A systematic review and meta-analysis
Source: Medicine (Baltimore). 2025 Jun 6;104(23):e42723. doi: 10.1097/MD.0000000000042723 (PMC12151038; doi:10.1097/MD.0000000000042723)
Supplement: Supplementary file 1 [file medi-104-e42723-s001.pdf]

Supplementary Table 1. Full texts not available.

Report	Title	Place of publication
Patil, 2022	Effectiveness of pelvic floor exercises as an adjunct to core stabilization on diastasis recti with lumbopelvic instability in postnatal women	Cochrane Central Register of Controlled Trials: CTRI/2022/06/043431
Simpson, 2021	After childbirth, is rectus abdominis training more effective than transversus abdominis training in reducing the gap between abdominal muscles in people with diastasis of the rectus abdominis muscles (DRAM)?	Cochrane Central Register of Controlled Trials: ACTRN12621000446864
Liyuan & Yan, 2021	Efficacy of acupuncture for women with Diastasis Recti Abdominis: randomized, Double-blind Controlled Trial	Cochrane Central Register of Controlled Trials: ChiCTR2100046959
Dangbeme & Kpadonou, 2021	What are the effective exercises after childbirth for the belly and the pelvic floor	Cochrane Central Register of Controlled Trials: PACTR202110679363984
Depledge, 2019	The effect of bracing on early postnatal women with Rectus Abdominis Diastasis	Cochrane Central Register of Controlled Trials ACTRN12619001638123
WHO, 2019	Physical therapy protocol on normal birth puerperium	Cochrane Central Register of Controlled Trials RBR- 6c3bvm
Stone, 2019	Impact of Exercise on Diastasis Rectus Abdominus	Cochrane Central Register of Controlled Trials NCT04049487
Rosmawati & Suhaila, 2019	Abdominal exercise for diastasis recti abdiminal (DRA)	Cochrane Central Register of Controlled Trials TCTR20190904005
Simpson, 2019	The effect of core muscle training vs. outer abdominal muscle training during the first three months postpartum on the size of the gap in abdominal muscles after birth in patients with diastasis of the rectus abdominis muscles	Cochrane Central Register of Controlled Trials: ACTRN12621000539831
Canarslan & Akbayrak, 2017	Assessing the effects of clinical pilates exercises on the strength of abdominal muscles and diastasis recti abdominis in pregnant women	Conference summary. v.28, n.2, p.S42-S43, 2017.
Kentrus, 2017	Abdominal Separation and the Female Core	IDEA Fitness Journal,v. 14, n.10, p.18-21, 2017
Kabir & Musa, 2017	Effect of therapeutic exercise in the reduction of postpartum diastasis recti.	Cochrane Central Register of Controlled Trials: PACTR201704002116256

Supplementary Table 2. Reports deleted after reading the full text.

**Study**	**Title**	**Publication**	**Exclusion Criteria**
Marini., 2016	The Influence of Pilates Soil on Separation of the Rectus abdominis muscle in Women in the Climatério	Clinical trial registration: RBR-2cfy62	Population
Mazzarino; Morris & Kerr, 2021	Pilates for low risk pregnant women: Study protocol for a randomized controlled trial	Journal of Bodywork and Movement Therapies, v.25, p.240–247, 2021.	Population
Cabral, 2021	Effects of exercise to prevent diastasis in pregnant women	Clinical trial registration: RBR-4v2r4bg	Population
Sunaeni & Isir, 2022	Demonstration of Gymnastics Pilates Muscle Tranversus Abdominis to Reduce Diastasis Recti	Bornova Izmir, v. 20, p. 2191 - 2198	Outcome
Preethi *et al.*, 2022	Effects of Kinesiotaping along with abdomen and pelvic floor exercise on diastasis recti abdominis for postpartum women in normal delivery versus LSCS	Biomedicine, v.42, n.1, p.143-147, 2022	Study Design
Theodorsen *et al.*, 2022	Effect of a specific exercise programme during pregnancy on diastasis recti abdominis: study protocol for a randomised controlled trial	BMJ Open, v.12, n.2, e056558, 2022	Study Design
Tseng, 2018	Acceptability and effectiveness of multi-media delivery of an exercise programme among postpartum women with lumbo pelvic pain in Taiwan	Clinical trial registration: ISRCTN51146251	Population

Supplementary Table 3. Characterization of articles included in the systematic review.

**Authors, year, and country**	**Nº participants (mean age and standard deviation)**	**Study duration, weekly frequency, and session duration**	**Abdominal exercise protocol**	**Other interventions**	**Control groups**	**Parity, type of birth, and postpartum time to start interventions**	**Equipment used to assess DRA**	**Results**
Sekar et al. (2024), India	44:<br>Hypopressive = 22 (27.2±1.7)<br>Isotonic abdominal = 22 (26.3±2.2)	8 weeks<br>3x week<br>(24 sessions)<br>20 minutes	Isotonic abdominal<br>4 exercises<br>Sets x reps: 3x20 (contraction 5 sec and relaxing 10 sec)<br>Effect control: NR	Hypopressive<br>5 hypopressive postures<br>Sets x reps: sets NR x 10 sec<br>Effect control: NR	None	Primiparous or multiparous<br>Vaginal or cesarean section<br>3 to 6 months	Caliper	Isotonic abdominal ↑ *vs.* Hypopressive
Elhosary et al. (2023), Egypt	40:<br>Isometric abdominal + low-calorie diet = 20 (30.1±2.9)<br>Isometric abdominal + tecar + low-calorie diet = 20 (30.4±3.4)	4 weeks<br>3x week<br>(12 sessions)<br>20 minutes	Isometric abdominal + low-calorie diet (1200 kcal/day)<br>3 exercises<br>Sets x reps: NR<br>Effect control: NR	Isometric abdominal + tecar + low-calorie diet (1200 kcal/day)<br>3 exercises<br>Sets x reps: NR<br>Effect control: NR<br>Tecar: 20 min; frequency 300 and 500	None	NR<br>Vaginal<br>NR	Ultrasound	Isometric abdominal + tecar + low-calorie diet ↑ *vs.* isometric abdominal + low-calorie diet
Moreira (2023), Brazil	44:<br>Hypopressive = 22 (34.4±5.5)<br>Control = 22 (31.8±2.9)	12 weeks<br>2x week<br>(24 sessions)<br>30 minutes	None	Hypopressive<br>(1ª-4ª week): 3-5 breaths<br>(5ª-8ª week): 2 breaths and 1 apnea<br>(9ª-12ª week): 1 breath and 1 apnea	Usual routine	NR<br>Vaginal or cesarean section<br>45 days and 6 months	Ultrasound	No difference

Kaya & Menek (2023), Turkey	45:<br>Isometric abdominal = 15 (30.6±3.5)<br>Isometric abdominal + AB = 15 (30.4±3.8)<br>AB = 15 (30.3±4.2)	8 weeks<br>3x week<br>(24 sessions)<br>Time NR	Isometric abdominal<br>Phase 1 and 2: 5 exercises<br>Phase 3: 3 exercises<br>Sets x reps: NR<br>Effect control: BORG (9-11; 11-12; 13-14)	Isometric abdominal + AB<br>Same protocol, just added the use of AB<br><br>Belt<br>(AB use only)	None	Primiparous or Multiparous<br>Vaginal or cesarean section<br>6 to 12 weeks	Ultrasound	Isometric abdominal + AB ↑ *vs.* Isometric abdominal<br><br>Isometric abdominal + AB ↑ *vs.* AB
Shohaimi *et al.* (2023), Malaysia	41:<br>Isometric abdominal + Isotonic = 21 (27±2.9)<br>Walking exercise = 20 (29±4)	8 weeks<br>3x week<br>(24 sessions)<br>Time NR	Isometric abdominal + Isotonic<br>Phase 1: 3 isometric<br>Phase 2 and 3: 2 isotonic, 1 isometric<br>Sets x reps: 3x10 (10sec phase 1 and 3sec on phase 2 and 3)<br>Effect control: NR	Walking exercises<br>Exercises: NR<br>Sets x reps: NR<br>Effect control: NR	None	Primiparous<br>Vaginal<br>2 to 4 months	Caliper	Isometric abdominal + isotonic abdominal ↑ *vs.* walking exercises
Yalfani *et al.* (2023), Iran	36:<br>Isometric abdominal = 12 (27.7±5.1)<br>Functional exercises = 12 (31.3±4.3)<br>Control = 12 (28.2±4.5)	8 weeks<br>3x week<br>(24 sessions)<br>50 minutes	Isometric abdominal<br>Sets x reps: NR<br>Effect control: NR	Functional exercises<br>Sets x reps: NR<br>Effect control: NR	Usual routine	Multiparous<br>Vaginal<br>2 to 4 months	Caliper	Isometric abdominal ↑ *vs.* control<br><br>Functional exercises ↑ *vs.* control
Gluppe *et al.* (2023), Norway	70:<br>Isotonic abdominal = 35 (35±4)<br>Control = 35 (33±3)	12 weeks<br>5x week<br>(60 sessions)<br>10 minutes	Isotonic abdominal<br>3 exercises<br>Sets x reps: 1-3 x 8-12<br>Effect control: NR	None	Usual routine	Primiparous or multiparous<br>Vaginal or cesarean section<br>6 to 12 months	Ultrasound	Isotonic abdominal ↑ *vs.* control

Lee *et al.* (2023), South Korea	35:<br>Isometric abdominal = 20 (34.1±2.9)<br>Control = 15 (33.8±3.9)	4 weeks<br>5x week<br>(20 sessions)<br>50 minutes	Isometric abdominal 8 core stabilization exercises from solo Pilates (3x a week) and 8 exercises at home, 2x a week)<br>Sets x reps: 1x 5-10 sec<br>Effect control: BORG (10-12; 12-14; 14-16 at the last week)	None	Usual routine	Primiparous<br>Vaginal or cesarean<br>2 to 12 months	Ultrasound	Isometric abdominal ↑ *vs.* control
Depledge *et al.* (2023), New Zealand	66:<br>Kinesiotaping = 33 (32.3)<br>AB = 33 (33.3)	8 weeks<br>(NR)	None	Kinesiotaping (taping on the diastasis region – daily to record the time used)<br><br>AB<br>(AB usage - daily to record the time used)	None	Primiparous or multiparous<br>Vaginal or cesarean section<br>72 hours after birth	Ultrasound	No difference
Simpson & Hahne (2023), Australia	44:<br>Isometric abdominal = 21 (31±4.5)<br>Isotonic abdominal = 23 (30±4.5)	6 weeks<br>10x week<br>(60 sessions)<br>Time NR	Isometric abdominal<br>1 exercise<br>Sets x reps: 1x10-20 (5 sec)<br>Effect control: NR	Isotonic abdominal<br>1 exercise<br>Sets x reps: 1x10-20 (5 sec)<br>Effect control: NR	None	NR<br>Vaginal<br>6-12 weeks	Caliper	Isotonic abdominal ↑ *vs.* Isometric abdominal
Kim *et al.* (2022), Switzerland	37:<br>Online isometric abdominal = 19 (31.6±3.9)<br>Off-line isometric abdominal = 18 (32.7±2.5)	6 weeks<br>2x week<br>(12 sessions)<br>40 minutes	Abdominal isométrico online<br>1 exercício<br>Sets x reps: 3-10 seg<br>Effect control: Borg (15)	Online isometric abdominal<br>1 exercise<br>Sets x reps: 3-10sec<br>Effect control: Borg (15)	None	NR<br>Vaginal or cesarean<br>6 to 12 months	Ultrasound	No difference

Liang *et al.* (2022), China	66:<br>Isotonic abdominal + NMES<br>= 33 (29.1±2.8)<br>Isotonic abdominal + PFMT + NMES = 33 (30±1.5)	6 weeks<br>3x week<br>(18 sessions)<br>20 minutes	Isotonic abdominal + NMES<br>4 exercises and contractions with NMES)<br>Sets x reps: 1x 15-20 (8-10 sec)<br>Effect control: NR<br>NMES: 3 contractions of 3 seconds and 5 seconds of relaxation	Isotonic abdominal + PFMT + NMES<br>4 abdominal exercises, PFMT and contractions with NMES)<br>Sets x reps: 1x 15-20 (8-10 sec)<br>Effect control: NR<br>NMES: 3 contractions of 3 seconds and 5 seconds of relaxation<br>PFMT NR	None	Primiparous or multiparous<br>Vaginal<br>6 months	Ultrasound	Isotonic abdominal + PFMT + NMES ↑ *vs.* Isotonic abdominal + NMES
Wei *et al.* (2022), China	32:<br>Isotonic abdominal + NMES<br>= 16 (33.4±0.3)<br>Control = 16 (35.1± 4.2)	6 weeks<br>7x week<br>(42 sessions)<br>Time NR	Isotonic abdominal + NMES<br>Number of NR exercises (abdominal exercise plus NMES)<br>Sets x reps: 1x20 (5sec)<br>Effect control: NR<br>NMES: NR	None	Usual routine	Primiparous or multiparous<br>Vaginal<br>6 months	Ultrasound	Isotonic abdominal + NMES ↑ *vs.* control

Liu *et al.* (2022), China	110:<br>Isotonic abdominal + PFMT + isometric + electroacupuncture = 54 (32.5± 4.2)<br>Isotonic abdominal + PFMT + isometric = 52 (32.7±3.5)	2 weeks<br>10x week<br>(20 sessions)<br>30 minutes	Isotonic abdominal + PFMT + isometric + electroacupuncture<br>Same protocol as group 2, but plus electroacupuncture<br>6 electroacupuncture points on the abdomen, intensity 4-6	Isotonic abdominal + PFMT + isometric<br>1 trunk flexion exercise, plus pelvic floor contraction and 3 core stabilization exercises<br>Set x reps: 1x10<br>Effect control: NR	None	Primiparous or multiparous<br>Vaginal or cesarean section<br>42 days to 1 year	Ultrasound	No difference
Safaee *et al.* (2022), Iran	45:<br>Isometric abdominal = 15 (28.6±3.2)<br>PFMT = 15 (30±2.3)<br>Control = 15 (29.9±3.9)	8 weeks<br>3x week<br>(24 sessions)<br>40-60 minutes	Isometric abdominal<br>18 core exercises and 4 dynamic lower limb stretches<br>Set x reps: 1x 6-10 (5-8 sec)<br>Effect control: NR	PFMT<br>Standing and sitting exercises (10 min warm-up with walking, stretching before PFMT)<br>Set x reps: 1x10-15 (3min interval)<br>Effect control: NR	Usual routine	Multiparous<br>Cesarean section<br>6 weeks	Caliper	Isometric abdominal and PFMT ↑ *vs.* control
Keshwani *et al.* (2021), Canada	32:<br>Isometric and isotonic abdominal = 6 (31±3)<br>Isometric abdominal, isotonic + AB = 8 (31±3)<br>AB = 6 (32±2)<br>Control = 7 (32±2)	12 weeks<br>7x week<br>(84 sessions)<br>Time NR	Isometric and isotonic abdominal<br>3 exercises: 1 transverse activation, 1 trunk flexion and 1 side plank<br>Set x reps: 3x10<br>Effect control: NR	Isometric abdominal, isotonic + AB<br>The same 3 exercises + use of AB<br>Set x reps: 3x10<br>Effect control: NR<br><br>AB<br>Using AB, taking it off only at bath time	Usual routine	Primiparous<br>Vaginal<br>3 weeks	Ultrasound	No difference

Laframboise *et al.* (2021), USA	8 (35.6±3.2):<br>Isometric abdominal + AB + PFMT (NR)<br>Control (NR)	12 weeks<br>3x week<br>(36 sessions)<br>Time NR	Isometric abdominal, AB, PFMT<br>Number of NR exercises (activation of the transverse, diaphragmatic breathing, AB and PFMT)<br>Set x reps: NR<br>Effect control: NR	None	Usual routine	Primiparous or multiparous<br>Vaginal or cesarean section<br>6 to 24 months	Caliper	Isometric abdominal + AB + PFMT ↑ *vs.* control
Pampolim *et al.* (2021), Brazil	50 (22.6±3.2):<br>Isometric abdominal + isotonic + PFMT = 25<br>Control = 25	2 interventions (6 hours and 18 hours postpartum)	Isometric abdominal + isotonic + PFMT<br>3 exercises (1 PFMT, 1 transverse contraction, 1 anterior trunk flexion)<br>Set x reps: 1x10 (service 1) - 20 (service 1)<br>Effect control: NR	None	Usual routine	Multiparous<br>Vaginal<br>6 hours	Caliper	Isometric abdominal + isotinic + PFMT ↑ *vs.* control
Ptaszkowska *et al.* (2021), Poland	24:<br>Kinesiotaping = 13 (27.5±5.8)<br>Control = 11 (27.6±4.4)	48-hour intervention	Kinesiotaping<br>Application of the technique on the transversus abdominis region	None	Application of a non-stretchable tape	Primiparous<br>Vaginal<br>6 weeks to 12 months	Caliper	Kinesiotaping ↑ *vs.* control

Awad *et al.* (2021), Egypt	50:<br>Isometric abdominal + AB = 25 (27±3.5)<br>AB = 25 (28.2±3.2)	8 weeks<br>3x week<br>(24 sessions)<br>Time NR	Isometric abdominal + AB<br>3 plank exercises, using AB<br>Set x reps: 3x20 (1min contraction and 2min relaxation)<br>Effect control: perceived effort (moderately intense)	AB<br>AB-use	None	NR<br>Vaginal<br>3 to 6 months	Ultrasound	Isometric abdominal + AB ↑ *vs.* AB
Saleem *et al.* (2021), Pakistan	40:<br>Isotonic abdominal + PFMT<br>= 20 (29.8±4.1)<br>Isometric abdominal + PFMT<br>= 20 (30.2±4.3)	6 weeks<br>3x week<br>(18 sessions)<br>Time NR	Isotonic abdominal + PFMT<br>4 exercises: abd flexion, glute strengthening, PFMT and spine strengthening)<br>Set x reps:3x10<br>Effect control: NR	Isometric abdominal + PFMT<br>(straight leg raise, glute strengthening, PFMT and spine strengthening)<br>Set x reps: 3x10<br>Effect control: NR	None	NR<br>Vaginal or cesarean<br>3 months	Palpation and Caliper	Isotonic abdominal + PFMT ↑ *vs.* Isometric abdominal + PFMT
Situt & Kanase (2021), India	40 (20-30 years):<br>Isometric abdominal + NMES<br>= 20<br>Isometric abdominal + Kinesiotaping<br>= 20	4 weeks<br>3x week<br>(12 sessions)<br>30 minutes	Isometric abdominal + NMES<br>7 abdominal exercises<br>Set x reps: 1x20 (5 seg)<br>Effect control: NR<br>NMES: in the rectus abdominis, frequency 80 pulses/min, pulse width 0.1-0.5ms	Isometric abdominal + Kinesiotaping<br>7 abdominal exercises plus Kinesiotaping<br>Set x reps: 1x20 (5 sec)<br>Effect control: NR	None	Primiparous<br>Vaginal<br>6 weeks	Caliper	Isometric abdominal + NMES ↑ *vs.* Isometric abdominal + Kinesiotaping

Botla & Saleh (2020), Egypt	36:<br>Isotonic abdominal + isometric = 18 (29.1±3.5)<br>Isotonic abdominal + isometric + NMES = 18 (29.5 ±3.5)	6 weeks<br>3x week<br>(18 sessions)<br>30-40 minutes	Isotonic abdominal + isometric<br>5 exercises (2 isometric and 3 isotonic)<br>Set x reps: 1x20 (contraction 5sec and relaxation 10sec)<br>Effect control: NR<br>(40 minutes)	Isotonic abdominal + isometric + NMES<br>5 exercises (2 isometric and 3 isotonic)<br>Set x reps: 1x20 (contraction 5sec and relaxation 10sec)<br>Effect control: NR<br>(30 minutes)<br>NMES: associated with exercise; frequency of 2,500Hz; pulse duration 200μs (protocol 10/50/10)	None	Multiparous<br>Vaginal<br>NR	Ultrasound	Isotonic abdominal + isometric + NMES ↑ *vs.* Isotonic abdominal + isometric

Yalfani *et al.* (2020)<br>Iran	24:<br>Isotonic abdominal + isometric+PFMT = 12 (29.7±4.9)<br>Control = 12 (28.2 ±4.5)	8 weeks<br>3x week<br>(24 sessions)<br>50-60 minutes	Isotonic abdominal + isometric + PFMT<br>Warm-up (10 min), Exercise (50 min), Cool-down (10 min)<br>Set x reps: NR<br>Effect control: NR	None	Usual routine	Multiparous<br>Vaginal<br>5 to 16 weeks	Caliper	Isotonic abdominal + isometric + PFMT ↑ *vs.* Control
Dave & Mahishale (2019), India	30:<br>Isotonic abdominal + isometric + AB = 15 (32.7±4.3)<br>Isotonic abdominal + isometric = 15 (29.6±2.4)	4 weeks<br>4-5x week<br>(16-20 sessions)<br>Time NR	Isotonic abdominal + isometric + AB<br>3 isometric, 1 isotonic and Superman + AB<br>Set x reps: 2-5x8-12 (10sec)<br>Effect control: light to moderate	Isotonic + isometric abdominal<br>3 isometric exercises, 1 isotonic and Superman<br>Set x reps: 2-5x8-12 (10sec)<br>Effect control: light to moderate	None	Primiparous or multiparous<br>Vaginal or cesarean section<br>1 to 3 years	Palpation and Caliper	Isotonic abdominal + isometric + AB ↑ *vs.* Isotonic abdominal + isometric
Thabet & Alshehri (2019), Saudi Arabia	30 (22-35 years old):<br>Isotonic + isometric abdominal = 20<br>AB + PFMT + isotonic and isometric abdominal = 20	8 weeks<br>3x week<br>(24 sessions)<br>Time NR	Isotonic + isometric abdominal<br>4 exercises (2 isotonic and 2 isometric)<br>Set x rep: 3x20 (5sec contraction and 10sec relaxation)<br>Effect control: NR	Belt + PFMT + isotonic abdominal + isometric abdominal<br>4 exercises (breathing exercises, PFMT, core stabilization and abd + AB)<br>Set x rep: 3x20 (5sec contraction and 10sec relaxation)<br>Effect control: NR	None	NR<br>Vaginal<br>3 to 6 months	Caliper	AB + PFMT + Isotonic abdominal and isometric ↑ *vs.* Isotonic abdominal + isometric

Izardi *et al.* (2018), Iran	32:<br>Isotonic abdominal = 17 (32.4±0.3)<br>Control = 16 (34.7±4.1)	6 weeks<br>7x week<br>(42 sessions)<br>Time NR	Isotonic abdominal<br>2 exercises<br>Set x rep: NR<br>Effect control: NR	None	Usual routine	Primiparous or multiparous<br>Vaginal<br>6 months	Ultrasound	Isotonic abdominal ↑ *vs.* Control
Bobowik & Dabek (2018), Poland	40:<br>Isometric abdominal + respiratory + functional = 20 (28.9±6.9)<br>Control = 20 (34.7±5.9)	6 weeks<br>7x week<br>(42 sessions)<br>Time NR	Isometric abdominal + respiratory + functional<br>Set x rep: NR<br>Effect control: NR	None	Usual routine	Primiparous or multiparous<br>Vaginal or cesarean section<br>0 to 3 days	Palpation	Isometric abdominal + respiratory + functional ↑ *vs.* Control
Gluppe *et al.* (2018), Norway	175:<br>Isotonic abdominal + PFMT<br>= 87 (29.5±4.3)<br>Control = 88 (30.1±4)	16 weeks<br>1x week<br>(16 sessions)<br>45 minutes	Isotonic abdominal + PFMT<br>Number of NR exercises (strengthening the abdomen, back, upper limbs, lower limbs, stretching, relaxation and PFMT)<br>Set x rep: NR<br>PFMT: 8-12 contractions, 6-8sec,<br>Effect control: NR	None	Usual routine	Primiparous<br>Vaginal<br>6 weeks to 12 months	Palpation	Isotonic abdominal + PFMT ↑ *vs.* Control

Tuttle *et al.* (2018), USA	33:<br>Isometric abdominal = 10 (31.2±4.9)<br>Kinesiotaping = 8 (31.8±3.7)<br>Isometric abdominal + Kinesiotaping = 5 (32.6±2.8)<br>Control = 7 (33±5.45)	12 weeks<br>5x week<br>(60 sessions)<br>NR	Isometric abdominal<br>4 stands<br>Set x rep: 1x10<br>Effect control: NR	Kinesiotaping<br>Application of Kinesiotaping (use for 4 to 7 days with a break of 2 to 4 days between applications)<br><br>Isometric Abdominal + Kinesiotaping<br>4 abdominal positions + application of Kinesiotaping (use for 4 to 7 days with a break of 2 to 4 days between applications)<br>Set x rep: 1x10<br>Effect control: NR	Usual routine	Primiparous or multiparous<br>NR<br>6 to 12 weeks	Ultrasound	Isometric abdominal + Kinesiotaping ↑ *vs.* control<br><br>Isometric abdominal + Kinesiotaping ↑ *vs.* Kinesiotaping<br><br>Isometric abdominal ↑ *vs.* Kinesiotaping<br><br>Isometric abdominal ↑ *vs.* control
Kamel & Yousif (2017), Bahrain	60:<br>Isotonic abdominal = 30 (29.5±3)<br>Isotonic abdominal + NMES = 30 (29.3±2.9)	8 weeks<br>3x week<br>(24 sessions)<br>30 minutes	Isotonic abdominal<br>4 ab and breathing exercises<br>Set x rep: 1x20<br>Effect control: NR	Isotonic abdominal + NMES<br>4 abd plus NMES exercises<br>Set x rep: 1x20<br>Effect control: NR<br>NMES: frequency 80 pulses/min, pulse width 0.1-0.5 mS and pulse rate 1-500p/s	None	Primiparous<br>Vaginal<br>2 months	Ultrasound	Isotonic abdominal + NMES ↑ *vs.* Isotonic abdominal

Walton *et al.* (2016), USA	9:<br>Isometric abdominal + isotonic + PFMT + AB = 5 (33±2.9)<br>Isotonic abdominal + PFMT + AB = 4 (32.7±3.8)	6 weeks<br>3x week<br>(18 sessions)<br>Time NR	Isometric abdominal + isotonic + PFMT + AB<br>Set x rep: 3x10 rep (plank: 10x10seg)<br>Effect control: NR	Isotonic abdominal + PFMT + AB<br>Set x rep:3x10 rep<br>Effect control: NR	None	Primiparous or multiparous<br>Vaginal or cesarean section<br>3 months to 3 years	Ultradound and Caliper	No difference
El-Mekawy *et al.* (2013), Egypt	30 (25-35 years):<br>Isotonic abdominal + isometric abdominal = 15<br>AB = 15	6 weeks<br>3x week<br>(18 sessions)<br>30 minutes	Isotonic abdominal + isometric abdominal<br>5 exercises: 3 isometric and 2 isotonic<br>Set x rep: 1x20 (5sec contraction and 10sec relaxation)<br>Effect control: NR	AB<br>AB use only during the day	None	Primiparous<br>Vaginal<br>2nd day to 6 weeks	Caliper	Isotonic abdominal + Isometric abdominal ↑ *vs.* AB
Mesquita *et al.* (1999), Brazil	50 (18-40 years):<br>Isometric abdominal + isotonic + PFMT = 25<br>Control = 25	6 hours and 18 hours postpartum (2 interventions)	Isometric abdominal + isotonic + PFMT<br>3 exercises: activation of the transverse abd, PFMT and isotonic contraction of the abd<br>Set x rep: 1x10-20<br>Effect control: NR<br>6h – 10 repetitions and 18h – 20	None	Usual routine	Primiparous or multiparous up to 4 children<br>Vaginal<br>6 hours	Caliper	Isometric abdominal + isotonic + PFMT ↑ *vs.* control

↑: significant intergroup improvement; Tecar: capacitive and resistive energy transfer; NMES: neuromuscular and muscular electrical stimulation; PFMT: pelvic floor muscle training; NR: not reported; AB: abdominal belt; Abd: abdominal
