# Supplementary material for: Effects of conservative approaches for treating diastasis recti abdominis in postpartum women: A systematic review and meta-analysis
Source: Medicine (Baltimore). 2025 Jun 6;104(23):e42723. doi: 10.1097/MD.0000000000042723 (PMC12151038; doi:10.1097/MD.0000000000042723)
Supplement: Supplementary file 2 [file medi-104-e42723-s002.pdf]

a)

Study or Subgroup	Abdominal exercise Mean [mm]	SD [mm]	Total	Control Mean [mm]	SD [mm]	Total	Weight	Mean Difference IV, Random, 95% CI
**5.3.1 Low risk of bias**								
Olupee et al. 2023	26	12	32	28	12	35	13.7%	-2.00 [-7.75, 3.75]
Keshwani et al. 2021	19.63	5.15	7	25.01	9.89	7	12.1%	-5.38 [-13.64, 2.88]
Lee et al. 2023	9.7	5.1	20	10	4.2	15	15.1%	-0.30 [-3.38, 2.78]
Tuple et al. 2018	12.2	3.2	10	13.3	2.3	7	15.2%	-1.10 [-3.71, 1.51]
**Subtotal (95% CI)**			**69**			**64**	**56.1%**	**-1.12 [-2.96, 0.72]**
Heterogeneity: Tau² = 0.00; Chi² = 1.38, df = 3 (P = 0.71); I² = 0%								
Test for overall effect: Z = 1.19 (P = 0.23)								
**5.3.2 High risk of bias**								
Bobowick & Dybek, 2018	4.04	2.3	20	16.75	7.01	20	15.0%	-12.71 [-15.94, -9.48]
Izardi et al. 2018	24	7	17	29	7	16	14.3%	-5.00 [-9.78, -0.22]
Safaee et al. 2022	25.6	6.5	15	46.5	4.6	15	14.6%	-20.90 [-24.93, -16.87]
**Subtotal (95% CI)**			**52**			**51**	**43.9%**	**-12.95 [-21.12, -4.79]**
Heterogeneity: Tau² = 47.79; Chi² = 25.34, df = 2 (P < 0.00001); I² = 92%								
Test for overall effect: Z = 3.11 (P = 0.002)								
**Total (95% CI)**			**121**			**115**	**100.0%**	**-6.82 [-12.82, -0.82]**
Heterogeneity: Tau² = 59.68; Chi² = 97.68, df = 6 (P < 0.00001); I² = 94%								
Test for overall effect: Z = 2.23 (P = 0.03)								
Test for subgroup differences: Chi² = 7.68, df = 1 (P = 0.006), I² = 87.0%								

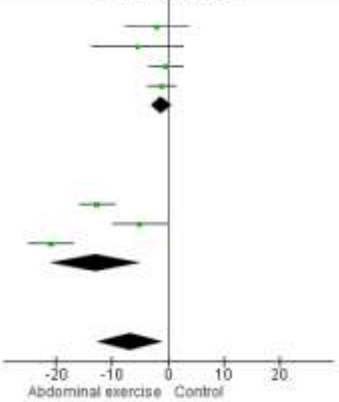

b)

Study or Subgroup	Abdominal exercise Mean [mm]	SD [mm]	Total	Control Mean [mm]	SD [mm]	Total	Weight	Mean Difference IV, Random, 95% CI
**10.2.1 Low risk of bias**								
Pampolim et al. 2021	33.9	2.8	25	34.6	2.8	25	53.4%	-0.70 [-2.25, 0.85]
**Subtotal (95% CI)**			**25**			**25**	**53.4%**	**-0.70 [-2.25, 0.85]**
Heterogeneity: Not applicable								
Test for overall effect: Z = 0.88 (P = 0.38)								
**10.2.2 High risk of bias**								
Mesquita et al. 1999	26.4	4.6	25	29.9	2.8	25	46.6%	-3.50 [-5.61, -1.39]
**Subtotal (95% CI)**			**25**			**25**	**46.6%**	**-3.50 [-5.61, -1.39]**
Heterogeneity: Not applicable								
Test for overall effect: Z = 3.25 (P = 0.001)								
**Total (95% CI)**			**50**			**50**	**100.0%**	**-2.00 [-4.74, 0.73]**
Heterogeneity: Tau² = 3.03; Chi² = 4.39, df = 1 (P = 0.04); I² = 77%								
Test for overall effect: Z = 1.44 (P = 0.15)								
Test for subgroup differences: Chi² = 4.39, df = 1 (P = 0.04), I² = 77.2%								

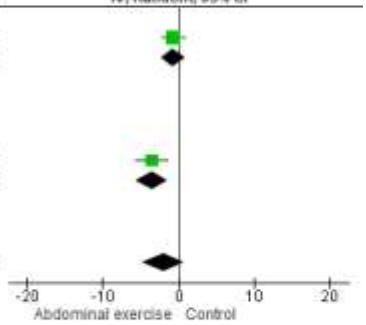

c)

Study or Subgroup	Isometric abdominal exercise Mean [mm]	SD [mm]	Total	Compressive methods Mean [mm]	SD [mm]	Total	Weight	Mean Difference IV, Random, 95% CI
**8.2.1 Low risk of bias**								
Keshwani et al. 2021	19.63	5.15	7	25.95	10.04	8	18.3%	-6.32 [-14.25, 1.61]
Tuple et al. 2018	12.2	3.2	10	15.3	2.2	8	41.1%	-3.10 [-5.60, -0.60]
**Subtotal (95% CI)**			**17**			**16**	**59.5%**	**-3.39 [-5.78, -1.01]**
Heterogeneity: Tau² = 0.00; Chi² = 0.58, df = 1 (P = 0.45); I² = 0%								
Test for overall effect: Z = 2.79 (P = 0.005)								
**8.2.2 High risk of bias**								
Kaya & Menek 2023	14.56	4.04	15	12.73	3.29	15	40.5%	1.83 [-0.81, 4.47]
**Subtotal (95% CI)**			**15**			**15**	**40.5%**	**1.83 [-0.81, 4.47]**
Heterogeneity: Not applicable								
Test for overall effect: Z = 1.36 (P = 0.17)								
**Total (95% CI)**			**32**			**31**	**100.0%**	**-1.69 [-6.03, 2.64]**
Heterogeneity: Tau² = 10.26; Chi² = 8.86, df = 2 (P = 0.01); I² = 77%								
Test for overall effect: Z = 0.77 (P = 0.44)								
Test for subgroup differences: Chi² = 8.28, df = 1 (P = 0.004), I² = 87.9%								

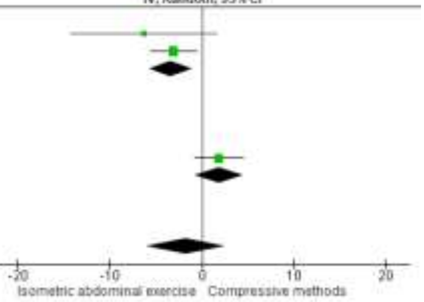

d)

Study or Subgroup	Abdominal exercises + interventions Mean [mm]	SD [mm]	Total	Control Mean [mm]	SD [mm]	Total	Weight	Mean Difference IV, Fixed, 95% CI
**4.2.1 Low risk of bias**								
Laframboise et al. 2021	24.4	11.5	4	20.4	6.6	4	63.7%	4.00 [-8.99, 16.99]
Tuple et al. 2018	11.9	2.9	5	13.3	23	7	36.3%	-1.40 [-18.63, 15.83]
**Subtotal (95% CI)**			**9**			**11**	**100.0%**	**2.04 [-8.33, 12.42]**
Heterogeneity: Chi² = 0.24, df = 1 (P = 0.62); I² = 0%								
Test for overall effect: Z = 0.39 (P = 0.70)								
**4.2.2 High risk of bias**								
**Subtotal (95% CI)**			**0**			**0**		**Not estimable**
Heterogeneity: Not applicable								
Test for overall effect: Not applicable								
**Total (95% CI)**			**9**			**11**	**100.0%**	**2.04 [-8.33, 12.42]**
Heterogeneity: Chi² = 0.24, df = 1 (P = 0.62); I² = 0%								
Test for overall effect: Z = 0.39 (P = 0.70)								
Test for subgroup differences: Not applicable								

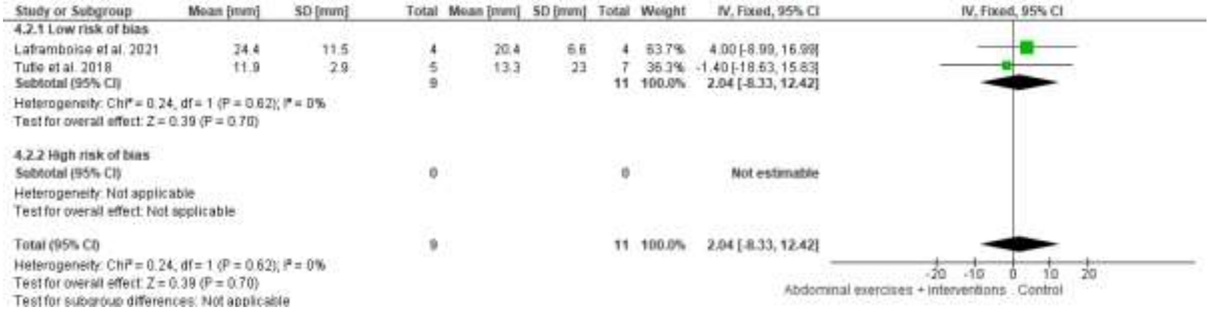

e)

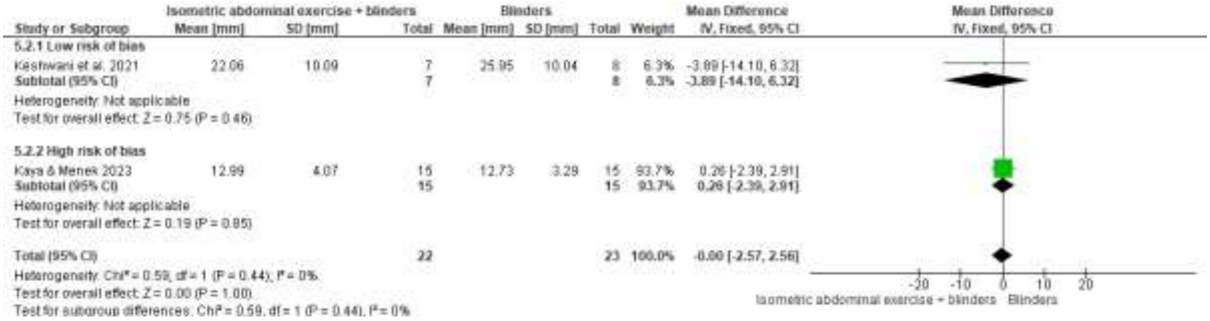

f)

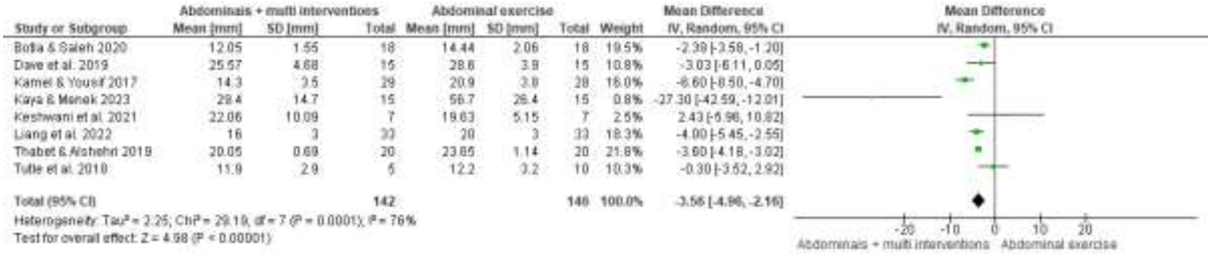

Supplementary Figure 1. Subgroup analysis (high and low risk of bias studies) of the effects of comparisons on abdominal diastasis: **(a)** abdominal exercises vs. control group; **(b)** abdominal exercises vs. control group (6h postpartum); **(c)** isometric abdominal exercises vs. compressive methods (abdominal belt and Kinesiotaping); **(d)** isometric abdominal exercises plus other interventions (abdominal belt, electrical stimulation or breathing exercises) Vs. control group; **(e)** isometric abdominal exercises plus abdominal belt Vs. abdominal belt; **(f)** abdominal exercises vs. abdominal exercises plus multi interventions (electrical stimulation, belt, breathing exercises, PFMT, Kinesiotaping).

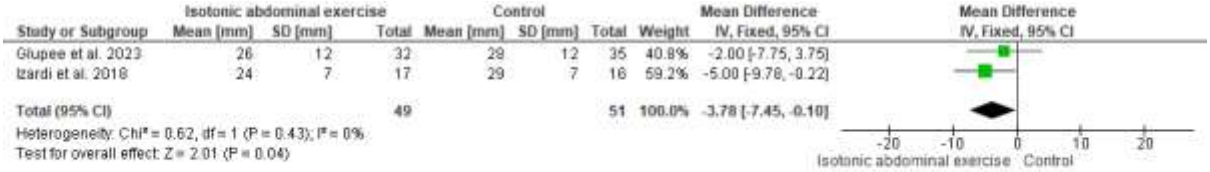

Supplementary Figure 2. Subgroup analysis of the effects of isotonic abdominal exercises vs control group on postpartum diastasis recti.

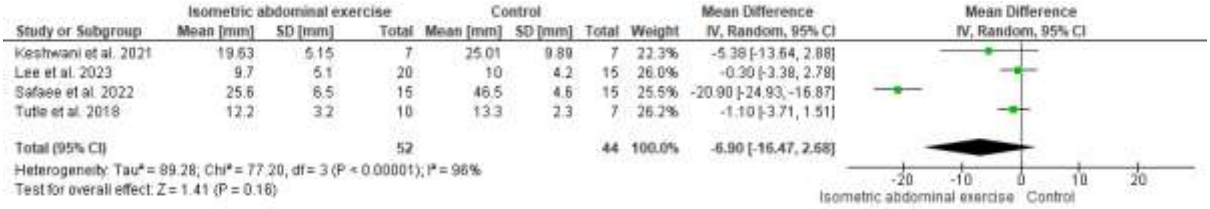

Supplementary Figure 3. Subgroup analysis of the effects of isometric abdominal exercises vs control groups on postpartum diastasis recti.

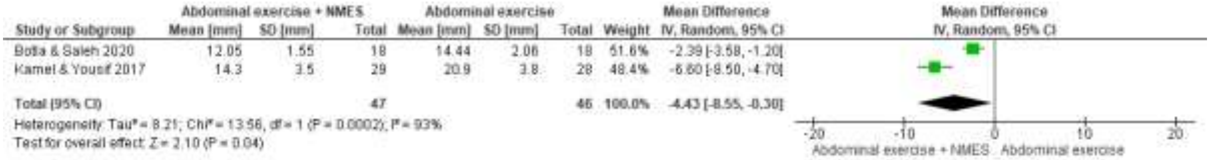

Supplementary Figure 4. Subgroup analysis of the effects of abdominal exercises vs abdominal exercises plus electrical stimulation on postpartum diastasis recti.
